# Clinicopathological correlations of perineural invasion in resected non-small cell lung cancer

**DOI:** 10.3389/fcell.2026.1872156

**Published:** 2026-07-22

**Authors:** Aleksandra Sejda, Dawid Sigorski, Jacek Józef Nowakowski, Ewa Iżycka-Świeszewska

**Affiliations:** 1 Department of Pathomorphology and Forensic Medicine, Collegium Medicum, University of Warmia and Mazury in Olsztyn, Olsztyn, Poland; 2 Department of Oncology, Collegium Medicum, University of Warmia and Mazury in Olsztyn, Olsztyn, Poland; 3 Department of Botany and Evolutionary Ecology, Faculty of Biology and Biotechnology, University of Warmia and Mazury in Olsztyn, Olsztyn, Poland; 4 Department of Pathology and Neuropathology, Medical University of Gdańsk, Gdańsk, Poland; 5 Department of Pathomorphology, Copernicus Hospital, Gdańsk, Poland

**Keywords:** neural niche, non-small cell lung cancer, perineural invasion, tumor microenvironment, tumor progression

## Abstract

**Background:**

The complex forms of the neural tumor microenvironment are involved in cancer progression and constitute the area of cancer neuroscience research. Perineural invasion (PNI), as one of the nerve-neoplastic interactions, is a well-known histopathological feature found in many solid malignancies, carrying potential prognostic significance. PNI frequency and prognostic significance in non-small cell lung cancer (NSCLC) remain poorly analyzed.

**Objective:**

This study aimed to evaluate the incidence of PNI in a cohort of NSCLC patients and its relationship with clinicopathological features and overall survival (OS).

**Material and Methods:**

The study population consisted of 124 archival, surgically resected NSCLC cases. Standard hematoxylin and eosin-stained tumor sections were assessed for the presence of PNI. Associations between PNI and clinicopathological parameters were analyzed, and its prognostic significance was evaluated in relation to overall survival (OS).

**Results:**

PNI was identified in 13.7% of cases, occurring more frequently in squamous cell carcinomas (21.0%) compared to adenocarcinomas (7.5%). It was significantly associated with hilar localization (p = 0.0005), squamous histology (p = 0.037), vascular invasion (p = 0.040), and lymph node metastases (p = 0.002). However, no significant association was found between PNI and overall survival (p = 0.216).

**Conclusion:**

PNI in NSCLC is an infrequent phenomenon. It shows a significant association with high-risk pathological features, including lymph node metastases and vascular invasion, suggesting a potential role in tumor progression. However, PNI was not significantly correlated with overall survival in this cohort of patients.

## 1 Introduction

The nervous system, as a crucial component of the tumor microenvironment (TME) may represent a missing link in the understanding of cancer development and progression (Monje et al., 2020; Restaino and Vermeer, 2022). In recent years, a growing number of studies have highlighted that tumor-associated nerves can influence the microenvironment through direct interactions with cancer cells within a specialized space known as the perineural niche (Bloomer et al., 2025; Winkler et al., 2023). These interactions can enhance vascularization, remodel the extracellular matrix and modulate the immune response, promoting tumor growth and progression through complex signaling pathways (Cui et al., 2023; Mravec, 2022; Hanahan and Monje, 2023). It offers new perspectives for developing targeted therapies focusing on disrupting these tumor-supportive neural networks (Li et al., 2022).

One of the most distinct morphological manifestations of this phenomenon is perineural invasion (PNI) (Restaino and Vermeer, 2022). It is now recognized not merely as a passive infiltration of malignant cells into nerve fibers but also as a complex, multistep process controlled by both cellular and molecular mechanisms (González-Castrillón et al., 2023; Chen et al., 2019; Liebig et al., 2009). Nevertheless, there is still a lack of consensus regarding its microscopic assessment, and discrepancies remain among pathologists in the diagnostic criteria for PNI. The most commonly accepted definition refers to cancer cell infiltration of any nerve layer or to a quantitative threshold suggesting at least 33% of the nerve fibers should be surrounded by tumor cells to confirm PNI. Yet, the morphological patterns of invasion are highly heterogeneous, including perineural or intraneural involvement, partial or complete (“crescent-like”) encirclement, sandwiching, lamellar (“onion-skin”) lamination, and tangential contact (Liebig et al., 2009; Ali et al., 2022). Most pathological protocols, such as those published by the College of American Pathologists, currently recommend a dichotomic evaluation of PNI as either present or absent (Lubig et al., 2018). However, several studies have shown that nerves may communicate with tumor cells prior to direct physical contact and that the tumor–nerve distance measured in histological sections may provide prognostic information beyond the conventional definition of PNI. Analyses of hematoxylin and eosin (H&E)–stained specimens have demonstrated that very small distances between tumor cells and nearby nerves are associated with worse clinical outcomes. Schmidt et al. reported that a tumor–nerve distance of ≤5 µm in head and neck squamous cell carcinoma (HNSCC) correlates with poorer survival (Schmitd et al., 2018). In this context, the current definition of PNI may require reconsideration.

The incidence of PNI varies significantly across different tumor types. The highest prevalence is noted in pancreatic adenocarcinoma, where reported frequencies are as high as 80% (Felsenstein et al., 2022; Gola et al., 2022). High rates are observed in prostate cancer and HNSCC, with PNI present in 60%–80% of tumors (Tao et al., 2024; Lee et al., 2010; Sigorski et al., 2021; Sejda et al., 2020). In contrast, in colorectal and breast carcinoma, the incidence ranges from 9% to 33% and from 1% to 25%, respectively, depending on the tumor subtype and histological grade (Wang et al., 2024; Knijn et al., 2016; Bahmad et al., 2025). Studies investigating PNI in other malignancies are underreported, with only a few papers focusing mainly on cholangiocarcinoma, gastric, vulvar, or cervical cancer. Similarly, limited data are available regarding the frequency of PNI in non-small cell lung cancer (NSCLC). A meta-analysis of 11 studies involving a total of 2,279 patients reported that PNI was present in approximately 9% of resected NSCLC cases, with individual study prevalence ranging from 4% to 31% (Liu et al., 2023).

Despite its variable prevalence, PNI is recognized as a marker of aggressive tumor behavior. Its presence is associated with an increased risk of local recurrence, distant metastasis, and reduced survival. In prostate cancer, PNI has been extensively studied and is a well-established histopathological predictor of biochemical recurrence, particularly in localized disease (Dell'Atti, 2016; Niu et al., 2022; Meng et al., 2015). Additionally, its presence correlates with extracapsular extension, higher Gleason score, and adverse pathological staging (Dell'Atti, 2016; Lee et al., 2010). In head and neck squamous cell carcinoma, PNI is an important determinant of increased risk of local relapse and shorter time to recurrence (Bakst et al., 2019). It is also linked to poorer prognosis, often necessitating more aggressive adjuvant therapy (Binmadi et al., 2023; Tao et al., 2024). A recently published systematic review with meta-analysis of 74 studies revealed that PNI-positive HNSCC patients had worse overall survival, disease-specific survival, and disease-free survival compared to those without PNI (Tao et al., 2024). Similarly, in gastric and cervical cancers, PNI has been shown to correlate with an unfavorable prognosis (Deng et al., 2014; Cui et al., 2015). In breast cancer, although less frequently encountered, PNI is emerging as a marker of poor prognosis, particularly in triple-negative and high-grade tumors (Bahmad et al., 2025).

Only a few data concerning PNI significance in NSCLC are available. They demonstrate the potential impact of PNI on worse survival rates (Liu et al., 2023; Sayar et al., 2004). Therefore, the aim of this study was to investigate the relationship between PNI and clinicopathological features in NSCLC and to evaluate its potential prognostic significance.

## 2 Materials and methods

### 2.1 Patient cohorts and pathologic examination

A total of 124 archival cases of resected non-small cell lung cancer (2011–2012) were retrospectively included in the study. Only primary adenocarcinomas (ADC) and squamous cell carcinomas (SCC) were evaluated. The study protocol was approved by the Institutional Bioethics Committee (No. 16/2023). The majority of patients underwent lobectomy, while a smaller subset were subjected to bilobectomy, pneumonectomy, or segmentectomy. Lymphadenectomy was performed in all but two cases. All tissue specimens were fixed in 10% neutral buffered formalin, embedded in paraffin and sectioned into 4 µm-thick slides. Tissue sampling was performed according to standard pathological protocols for lung cancer specimens, with multiple blocks routinely obtained from the tumor and adjacent lung parenchyma, ensuring representative histological assessment. Depending on the tumor diameter, one sample per 1 cm of tumor size was obtained for routine histopathological examination. At least two formalin-fixed, paraffin-embedded (FFPE) blocks were prepared per case (mean: 4 blocks). All slides were evaluated using standard H&E staining, without additional ancillary studies for identification of nerve fibers. Tumors were reclassified according to the most recent edition of the WHO Classification of Thoracic Tumors. Pathological stage was reassessed based on the 8th edition of the American Joint Committee on Cancer/Union for International Cancer Control (AJCC/UICC) staging guidelines. Overall survival (OS) was defined as the time from surgical resection to death or last follow-up.

### 2.2 PNI evaluation

PNI was defined as the presence of cancer cell infiltration in any nerve layer, regardless of the invasion pattern. Nerve fibers were identified as rounded structures with a diameter between 0.15 and 0.35 mm typically located in close association with vessels within neurovascular bundles. They were recognized on H&E staining by their characteristic fascicular architecture, wavy appearance, elongated Schwann cell nuclei, and a distinct perineurial layer. All available whole sections from each tumor were viewed under a light microscope at 100×, 200×, and 400× magnification to assess for perineural invasion. PNI was recorded as positive if infiltration of at least one nerve fiber was observed; otherwise, it was considered negative. In addition, the total number of affected nerves was quantified. All cases with regard to pathological features, including PNI status, were independently reviewed by two pathologists who were blinded to survival outcomes during slide evaluation, and any discrepancies were resolved by consensus discussion.

### 2.3 Statistical analysis

Relationships between PNI and gender, tumor localization, histological type, grade, vascular invasion, and STAS were tested using Fisher’s exact test. The association between PNI and ADC subclassification, type of pleural invasion, pT, pN, AJCC stage, and type of surgical resection were evaluated using the likelihood ratio chi-square test G^2^ or Fisher’s exact test, depending on the expected number of subclasses to be separated, in accordance with the assumptions for the test’s function. Given the small number of cases in certain subgroups, all subgroup analyses should be interpreted as exploratory. The Kaplan-Meier method was used to estimate overall survival time. Comparison of the slopes of the survival curves was tested using the Mantel-Cox log-rank test. Due to the limited number of PNI-positive cases, multivariable analysis was not performed. All statistical analyses were performed using SPSS version 29.0.

## 3 Results

### 3.1 Patients characteristics

The study cohort consisted of 124 patients, including 80 males (64.5%) and 44 females (35.5%). The median age of the participants was 61 (range: 48–81 years). The majority of patients underwent lobectomy (n = 109; 87.9%). Adenocarcinoma was the slightly more prevalent histological subtype, identified in 67 cases (54.0%), compared to squamous cell carcinoma, which was diagnosed in 57 cases (46.0%). The median tumor diameter was 3.3 cm (range: 0.7–12 cm), with a median size of 4.0 cm for SCC and 2.8 cm for ADC. Tumor localization was nearly evenly distributed, with 63 cases (50.8%) located peripherally and 61 cases (49.2%) in the hilar region. The majority of tumors demonstrated intermediate differentiation (grade 2), accounting for 73 cases (58.9%), while the remaining 51 cases (41.1%) were poorly differentiated (grade 3). Among adenocarcinomas, the most common histological growth patterns were acinar and solid, found in 38 cases (56.7%) and 24 cases (35.8%), respectively. STAS were identified in 37 (30%) of cases, and vascular invasion in 39 (31.5%). The number of cases without pleural involvement (n = 58, 46.8%) was comparable to those with pleural infiltration, with the PL1 pattern being the most frequently observed (n = 60, 48.4%). Almost half of the patients were classified as stage pT2a (n = 53; 42.7%), followed by pT3 in 22 cases (17.8%) and pT1c in 16 cases (12.9%). The remaining T categories were less frequently observed. Lymph node metastases were present in 44 cases, including 31 cases (25.0%) with N1 and 13 cases (10.5%) with N2 involvement. The most common AJCC/UICC stage was IB (n = 35; 28.3%), followed by stage IIIA, observed in 31 patients (25.0%).

### 3.2 PNI incidence and morphologic characteristic

Perineural invasion was found in 17 tumors (13.7%), including 5 adenocarcinomas (7.5%) and 12 squamous cell carcinomas (21.0%), occurring more frequently in male patients (n = 13; 16.2%). In 7 cases, a single nerve fiber was involved, 6 of which were squamous cell carcinoma. In another 7 cases, two nerve fibers were affected (3 SCC and 4 ADC). Additionally, single cases exhibited infiltration of three, four, and more than ten nerve fibers.

**FIGURE 1:**
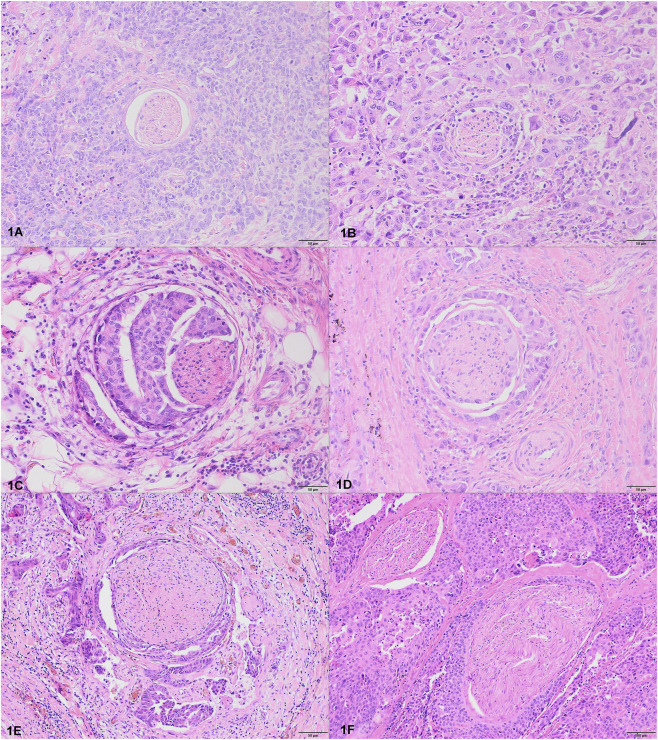
Examples of perineural invasion patterns in non-small cell lung cancer. Whole nerve fibers embedded in the tumor mass in poorly differentiated squamous cell carcinoma (**(A)**, 20x) and solid adenocarcinoma (**(B)**, 20x). Crescent-like pattern (**(C)**, 20x) and onion-skin lamellation (**(D)**, 20x) in ADC. PNI involvement of the nerve fibers located in the hilum in close proximity to the tumor front in SCC (**(E)**, 20x). Nerve fiber partially encircled by SCC tumor cells; upper left, tangential tumor–nerve contact without direct infiltration (**(F)**,20x).

The most frequently observed pattern of perineural invasion was complete circumferential encirclement of the nerve fibres, occurring in two distinct forms. In some cases, the nerve was entirely embedded within the tumor mass, while in others, the involved nerve fibres were located at some distance from the tumor front. Additionally, a crescent-like pattern of infiltration was observed in two cases, and in one case, a lamellar, onion-skin like lamellation was present. Partial involvement, as well as tangential contact, was also identified, but only in a minority of cases. In the cases with multiple PNI involvement, several distinct patterns coexisted. Representative examples of each infiltration pattern are presented in Figure 1. Most infiltrated nerves were located in the hilar or perihilar region, while others in close proximity to the bronchi and angiovascular bundles, which is consistent with the anatomical distribution of peripheral nerves in the lungs. There was no involvement of pleural nerves.

### 3.3 Clinicopathological correlations of PNI

The median diameter of PNI positive tumors was 4.3 cm (range: 2,3-7,1 cm). Among tumors with perineural invasion, 15 cases (24.6%) were located in the hilar region, while only 2 cases were found in the more peripheral parts of the lung. With respect to tumor differentiation, 10 cases (13.7%) were classified as grade 2, and the remaining 7 cases (13.7%) as grade 3. Simultaneous PNI and vascular invasion was observed in 9 (23.1%) out of 39 (31.5%) VI positive cases. Additionally, only 3 (8.1%) patients with PNI had STAS too.

The presence of PNI was significantly associated with hilar localization (p = 0.0005), squamous cell histology (p = 0.037), lymph node metastases (p = 0.002) and vascular invasion (p = 0.040). PNI was detected most frequently in patients after lobectomy compared to patients after other techniques (p = 0.0006). However, these associations should be interpreted cautiously, as they may be confounded by tumor stage, size, and localization. There was no relationship between PNI-positivity and gender (p = 0.413), ADC subclassification (p = 0.379), pleural invasion (p = 0.812), grade (p = 0.999), STAS (p = 0.392), pT (p = 0.522), and AJCC stage (p = 0.536). The detailed data of the presence of PNI in subgroup patients depends on the distinguishing category and clinicopathological features, and the results of the tested relationships are summarized in Table 1.

**TABLE 1:** Patient characteristics and clinicopathological correlations with perineural invasion in non-small cell lung cancer. PNI–perineural invasion; SCC–squamous cell carcinoma; ADC–adenocarcinoma; STAS–spread through air spaces; PL–pleural invasion.

Characteristics	Study cohort	Presence of PNI		
	n (%)	PNI positive n (%)	PNI negative n (%)	P value
Gender	​	​	​	0.413
Male	80 (64.5)	13 (16.2)	67 (83.8)	​
Female	44 (35.5)	4 (9.1)	40 (90.9)	​
Tumor localization	​	​	​	**0.0005**
Peripheral	63 (50.8)	2 (3.2)	61 (96.8)	​
Hilar	61 (49.2)	15 (24.6)	46 (75.4)	​
Histology	​	​	​	**0.037**
ADC	67 (54.0)	5 (7.5)	62 (92.5)	​
SCC	57 (46.0)	12 (21.0)	45 (79.0)	​
ADC subclassification	​	​	​	0.379*
Acinar	38 (56.7)	4 (10.5)	34 (89.5)	​
Micropapillary	3 (4.5)	1 (33.3)	2 (66.7)	​
Papillary	2 (3.0)	0 (0.0)	2 (100.0)	​
Solid	24 (35.8)	0 (0.0)	24 (100.0)	​
Grade	​	​	​	0.999
2	73 (58.9)	10 (13.7)	63 (86.3)	​
3	51 (41.1)	7 (13.7)	44 (86.3)	​
Pleural invasion	​	​	​	0.812*
PL0	58 (46.8)	9 (15.5)	49 (84.5)	​
PL1	60 (48.4)	7 (11.7)	53 (88.3)	​
PL2	1 (0.8)	1 (100.0)	0 (0.0)	​
PL3	5 (4.0)	0 (0.0)	5 (100.0)	​
STAS	​	​	​	0.392
Present	37 (30,0)	3 (8.1)	34 (91.9)	​
Absent	87 (70.0)	14 (16.1)	73 (83.9)	​
Vascular invasion	​	​	​	**0.040**
Present	39 (31.5)	9 (23.1)	30 (76.9)	​
Absent	85 (68.5)	8 (9.4)	77 (90.5)	​
pT	​	​	​	0.522*
1a	1 (0.8)	0 (0.0)	1 (100.0)	​
1b	9 (7.2)	0 (0.0)	9 (100.0)	​
1c	16 (12.9)	2 (12.5)	14 (87.5)	​
2a	53 (42.7)	6 (11.3)	47 (88.7)	​
2b	11 (8.9)	3 (27.3)	8 (72.7)	​
3	22 (17.8)	4 (18.2)	18 (81.8)	​
4	12 (9.7)	2 (16.7)	10 (83.3)	​
pN	​	​	​	**0.002** *
0	78 (62.9)	5 (6.4)	73 (93.6)	​
1	31 (25.0)	11 (35.5)	20 (64.5)	​
2	13 (10.5)	1 (7.7)	12 (92.3)	​
No data	2 (1.6)	0 (0.0)	2 (100.0)	​
AJCC stage	​	​	​	0.536*
Ia	21 (16.9)	1 (4.8)	20 (95.2)	​
Ib	35 (28.3)	3 (8.6)	32 (91.4)	​
IIa	18 (14.5)	3 (16.7)	15 (83.3)	​
IIb	16 (12.9)	4 (25.0)	12 (75.0)	​
IIIa	31 (25.0)	6 (19.4)	25 (80.6)	​
IIIb	1 (0,8)	0 (0.0)	1 (100.0)	​
No data	2 (1.6)	0 (0.0)	2 (100.0)	​
Type of surgical resection	​	​	​	**0.0006** *
Lobectomy	109 (87.9)	11 (10.1)	98 (89.9)	​
Bilobectomy	5 (4.1)	2 (40.0)	3 (60.0)	​
Pulmonectomy	6 (4.8)	4 (66.7)	2 (33.3)	​
Segmentectomy	4 (3.2)	0 (0.0)	4 (100.0)	​

* Groups were combined for testing: ADC, subclassification–acinar vs. (papillary, micropapillary, solid); Pleural invasion–PL0, vs. (PL1-3); pT – (pT1a-c) vs. (pT2a,b, pT3, pT4); pN–pN0, vs. (pN1-2); AJCC, stage - stage I-II, vs. III); Type of surgical resection–lobectomy vs. (bilobectomy, pulmonectomy, segmentectomy).

Bold values indicate statistically significant differences (p < 0.05).

### 3.4 Survival analysis

**FIGURE 2:**
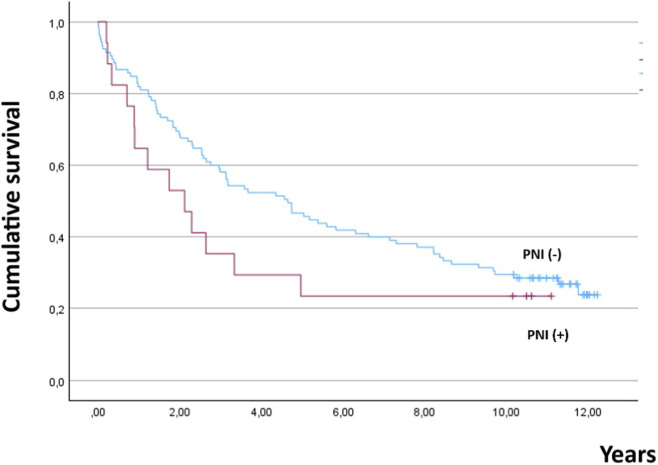
Kaplan–Meier curves for overall survival according to perineural invasion (PNI) status in 124 patients with NSCLC who underwent surgical treatment.

For the entire group, the median follow-up time was 3.45 years (range: 0–12.2), and median survival time was 3.58 years 92 (74,2%) patients died during the time of observation. The median overall survival was 2.12 years (95% CI: 0.67–3.57) in patients with perineural invasion, compared with 4.64 years (95% CI: 2.81–6.47) in PNI-negative cases. The median overall survival for the entire cohort was 3.58 years (95% CI: 2.21–4.95). However, the difference did not reach statistical significance (log-rank test, p = 0.216). Kaplan–Meier survival curves according to PNI status are shown in Figure 2.

## 4 Discussion

PNI has been rarely studied in non-small cell lung cancer, despite its well-established prognostic value in several other solid tumors. There are limited data describing this phenomenon in NSCLC. Given the aggressive nature of these malignancies and the ongoing search for reliable histopathological prognostic markers, investigating the role of PNI in this context is both timely and clinically relevant. Our study provides insight into the incidence, clinicopathological associations, and prognostic significance of perineural invasion in a set of 124 patients with resected NSCLC. Our assessment is based on diagnostically routine H&E staining, and we did not perform additional ancillary studies to confirm the presence of nerve fibers. In our cohort, PNI was identified in 13.7% of cases. This finding is consistent with previously published studies, where the reported PNI incidence fluctuated from 2% to 30% (Demir et al., 2008; Kiliçgün et al., 2010; Sayar et al., 2004; Yilmaz et al., 2011). A key factor contributing to the variability in PNI rates across studies may be the lack of a standardized definition of PNI. Not without significance are differences in the examined sample size, especially since the exact prevalence is challenging to determine due to diagnostic variability and the need for meticulous histopathological examination. In addition, biological factors may also contribute to the observed variability and relatively low prevalence of PNI in lung cancer. Although PNI is often considered to arise in part due to anatomical proximity, it is increasingly recognized as a result of complex bidirectional interactions between cancer cells and nerves, mediated by multiple molecular mechanisms and epigenetic changes. Numerous cytokines, growth and neurotrophic factor–dependent paracrine and endocrine signaling pathways contribute to tumor–nerve crosstalk and may influence the extent of PNI observed in individual cases (Wang et al., 2025; Wang et al., 2020; Chen et al., 2019).

In prior studies, correlations between PNI and other clinicopathological parameters in NSCLC have been poorly explored. Our data showed that PNI was more frequently observed in tumors after lobectomy with squamous cell histology, particularly those situated in the hilar region. In contrast to our findings, Yılmaz et al. highlighted a correlation only between PNI and higher histological grades (grade 2 and 3), while Moon et al. demonstrated a significant association between the presence of PNI and extensive tumor necrosis (Moon et al., 2022; Yilmaz et al., 2011). Notably, we observed a significant association between PNI and both vascular invasion and lymph node metastasis. These factors are well-established contributors to more aggressive clinical behavior in malignancies. In a meta-analysis conducted by Wang et al., vascular invasion was identified as an independent prognostic factor for overall survival and recurrence-free survival in lung cancer (Wang et al., 2011). Similar to PNI, VI warrants further investigation to determine its prognostic value and its potential implications for TNM staging. Lymph node metastases are widely recognized as indicators of poor prognosis. Our findings reinforce the potential link between PNI and increased tumor aggressiveness, particularly its association with the occurrence of lymph node metastases. In gastric cancer, the presence of combined LVI/PNI emerged as an independent risk factor for lymph node metastasis and for poorer overall survival (Zhang et al., 2023). The predictive role of PNI in metastatic disease should be further explored in future studies. In a previous studies contributing to the evaluation of prognostic role of vascular and perineural invasion, no relationship between these two features was analyzed (Yilmaz et al., 2011; Sayar et al., 2004).

PNI has been indicated to correlate with more aggressive behavior in many malignancies, although the data related to NSCLC are very limited. Our results demonstrated that the association between PNI and prognosis did not achieve statistical significance. Given the relatively small number of PNI-positive cases, our study may have been underpowered to detect survival differences. However, in several studies, the impact of neural invasion on patients survival was proved (Tran et al., 2013). In a meta-analysis concerning the prognostic significance of PNI in NSCLC, a poorer outcome was suggested (Liu et al., 2023). Some studies described PNI as an unfavorable prognostic indicator in resected NSCLC (Sayar et al., 2004; Kiliçgün et al., 2010). One of the studies examining 108 patients with locally advanced N2-positive NSCLC reported PNI as a negative prognostic factor for disease-free survival (Hsieh et al., 2015). Similar findings were demonstrated in the study of Demir et al. were PNI affected outcome in a set of T3 resected patients (Demir et al., 2008). Patients with stage IIIA tumors and perineural invasion had a statistically significantly poorer survival than those without PNI (Kiliçgün et al., 2010). In a group of patients over 70 years, PNI was confirmed as an adverse negative prognostic factor of long-term cancer-related mortality (Tantraworasin et al., 2020). On the contrary, there are some contradictory data suggesting a negative or no correlation between PNI and patient outcome (Poncelet et al., 2008; Yilmaz et al., 2011). Single study revealed that patients with perineural invasion are more likely to develop brain metastases (Sun et al., 2021).

In our work we focused on H&E stains, where nerve fibers are relatively easy to assess without additional immunostains. However, it is worth noting that there are data on the investigation of very small nerves and nerve density in tumor specimens (Schmitd et al., 2021). In prostate cancer, studies have demonstrated that increased nerve density within tumor areas and in adjacent stroma is associated with markers of aggressive behavior (Sigorski et al., 2021). Data on this topic in NSCLC remain limited. One study reported that sympathetic nerve fibers were predominantly located in the paratumoral area, whereas parasympathetic fibers were mainly confined to the tumor tissue and both correlated with survival (Shao et al., 2016). Although immunohistochemical markers such as S100 or SOX10 may increase sensitivity in the detection of small nerve fibers, particularly within the tumor stroma, their use in every case is not feasible in daily diagnostic workflow due to practical and resource-related limitations. It should be acknowledged that the exclusive reliance on H&E staining may potentially lead to underdetection of very small nerve involvement, and therefore represents a methodological limitation of the present study. However, this approach reflects real-world diagnostic conditions and enhances the clinical applicability and translational relevance of our findings. Importantly, the majority of published studies investigating PNI in solid tumors, including non-small cell lung cancer, are also based on H&E morphology, which allows for comparability of results across studies (Dell'Atti, 2016; Poncelet et al., 2008, Yilmaz et al., 201). In this context, our findings should be interpreted within the framework of standard histopathological practice, while acknowledging the inherent limitations of H&E-based assessment.

This study is one of the few publications assessing PNI clinicopathological correlations together with its prognostic significance in NSCLC. The basic limitation of this study is the relatively small population of patients. The relatively low prevalence of PNI in NSCLC may reflect both biological factors, such as variable tumor–nerve interactions and differences in intrapulmonary nerve distribution, as well as technical aspects related to sampling and detection in routine histopathological assessment. Nevertheless, our findings are intriguing and highlight the need for further investigation in larger cohorts.

## 5 Conclusion

The prognostic significance of perineural invasion in non-small cell lung cancer remains unclear. It represents a relatively infrequent phenomenon in NSCLC. However, our findings suggest that PNI may contribute to increased lymph node involvement. Standardized criteria for identifying and reporting PNI are essential for accurately assessing its prognostic value. Many authors emphasize the lack of a uniform definition and consistent reporting of PNI as a major challenge that requires systematic resolution. Further research is needed to clarify PNI’s prognostic importance in NSCLC, its potential role in guiding treatment decisions, and its utility in enhancing the prognostic accuracy of the TNM staging system.

## Data Availability

The datasets presented in this article are not readily available because no permision to share data. Requests to access the datasets should be directed to aleksandra.sejda@uwm.edu.pl.
